# Supplementary material for: Evaluation of the Impact of Catheter Ablation Procedure on Outcomes and Economic Burden in Patients with Atrial Fibrillation: Real-World Data from Italian Administrative Databases
Source: Healthcare (Basel). 2022 Dec 17;10(12):2561. doi: 10.3390/healthcare10122561 (PMC9778702; doi:10.3390/healthcare10122561)
Supplement: Supplementary file 1 [file healthcare-10-02561-s001.zip › healthcare-2057419-supplementary.pdf]

Supplementary material

Supplementary Table S1. Characteristics of AF patients with and without trans-catheter ablation procedure, post PSM.

	**Patients with ablation**	**Patients without ablation**	**P-value**	**SMD**
Patients, n	3,067	18,402	-	
Age (years) at ablation procedure, mean (SD)	63.1 (12.4)	67.3 (12.9)	<0.001	0.333
Males, n (%)	2,064 (67.3)	11,572 (62.9)	<0.001	0.093
Tumors, n (%)	38 (1.2)	283 (1.5)	0.207	0.026
COPD, n (%)	703 (22.9)	4,585 (24.9)	0.018	0.047
Diabetes mellitus, n (%)	434 (14.2)	3,082 (16.7)	<0.001	0.072
CKD, n (%)	102 (3.3)	610 (3.3)	0.975	0.001
Hyperthension, n (%)	2,631 (85.8)	15,981 (86.8)	0.013	0.047
Antiarithmics, n (%)	2,259 (73.7)	13,061 (71.0)	0.002	0.060
Beta-blockers, n (%)	2103 (68.6)	12,420 (67.5)	0.238	0.023
Calcium channel blockers, n (%)	708 (23.1)	4,787 (26.0)	0.001	0.068
Anticoagulants, n (%)	2,642 (86.1)	15,882 (86.3)	0.808	0.005
Coronary artery disease hospitalizations, n (%)	240 (7.8)	1,408 (7.7)	0.738	0.007
Cerebrovascular disease hospitalizations, n (%)	97 (3.2)	639 (3.5)	0.383	0.017
Heart failure hospitalizations, n (%)	457 (14.9)	2,145 (11.7)	<0.001	0.096
Peripheral vascular disease hospitalizations, n (%)	19 (0.6)	133 (0.7)	0.528	0.013
PTCA hospitalizations, n (%)	92 (3.0)	484 (2.6)	0.241	0.022

CKD, chronic kidney disease; COPD, chronic obstructive pulmonary disease; PSM, propensity score matching; PTCA, percutaneous coronary angioplasty; SD, standard deviation; SMD, standardized mean difference.
